# Supplementary material for: Membrane-anchored PrPSc is the trigger for prion synaptotoxicity
Source: PLoS Pathog. 2026 Jan 28;22(1):e1013911. doi: 10.1371/journal.ppat.1013911 (PMC12867327; doi:10.1371/journal.ppat.1013911)
Supplement: Attachment — Submitted filename: Response to Reviewers.pdf [file ppat.1013911.s011.pdf]

Rebuttal Letter – Response to Reviewers

December 30, 2025

The Editors
PLOS Pathogens

PPATHOGENS-D-25-01703 (Gatdula et. al)

We greatly appreciate each of the reviewers' favorable comments on our manuscript, and their constructive suggestions for improving it. We have been able to address each of the points raised by the reviewers, as detailed below. The revised manuscript includes new data (Fig 1D, Fig 3B, Fig 3C, Fig S7), which we believe significantly strengthen the impact of the study. We hope the revised manuscript will now be acceptable for publication.

An additional author (**Jose Andres Alepuz Guillen**) has been included in the revised manuscript, and the present address of one of the authors: (**Robert C.C. Mercer**), has been added.

This rebuttal letter contains one figure that we included to address a major point raised by reviewers #2 and #3 concerning the use of anchorless PrP constructs, but which we did not think warranted inclusion in the revised paper.

PART II – MAJOR ISSUES

Reviewer #1:

"It remains unclear how Figure S6 relates to the overall finding of the study as expression of wild-type PrP in hippocampal neurons of Prnp$^{-/-}$ mice challenged with 22L prions does not yield visible puncta, in contrast to the images presented in Figure 3. If limited magnification accounts for the absence of puncta, this should be explicitly stated, and higher-magnification images should be provided. Figure 3 shows puncta in hippocampal neurons from Prnp$^{-/-}$ mice reconstituted with wild-type, but not in those expressing G126V PrP. While this finding is consistent with results obtained from the propagation model, the statistical power of the experiment is limited ("10 neurons from 2 independent experiments were visualized in randomly selected areas"). A larger number of independent replicated experiments is recommended to support the conclusion that PrP conversion is a prerequisite for synaptotoxicity."

Response:

Figure S6 serves three purposes:

(1) It shows raw images before processing by Gaussian blurring, as detailed in the Methods section (lines 432-448), to produce images used to quantify PrP^{Sc} puncta (shown in Figure 3).
(2) Because lentiviral transduction is sometimes not fully efficient, the figure confirms successful expression of either WT PrP or G126V PrP in transduced Prnp$^{-/-}$ neurons.
(3) It demonstrates that uninfected (–22L) neurons express PrP^{C} at the cell surface in the absence of phosphatidylinositol-specific phospholipase C (PI-PLC), whereas PI-PLC treatment eliminates detectable surface PrP^{C}. This validates that the PI-PLC incubation conditions used are sufficient to remove cell-surface PrP^{C} from neurons.

We agree with the reviewer that the statistical power of the experiment in the original submission is limited (n=2). To address this, we performed the experiment two more times, yielding a total of at least 20 neurons from 4 independent experiments. This new set of data has been integrated into the revised manuscript (Figure 3 B, C). The new statistical analysis has also been updated in the legend to Figure 3.

Reviewer #2:

"To more precisely demonstrate that synaptotoxicity is dependent on membrane-anchored PrP^{Sc}, it would be valuable to include hippocampal neurons expressing GPI-anchorless wild-type PrP in the assay. This parallel experiment would help clarify whether membrane anchorage is indeed necessary for the observed toxic effects."

Response:

We agree with the reviewer that infecting neurons that express anchorless versions of PrP (GPI- PrP) would strengthen our conclusions. In fact, we have experimented extensively with expressing anchorless forms of PrP (WT and G126V) in N2a cells. However, these forms are expressed at very low levels in cultured cells and are virtually undetectable in the culture medium. Low expression of anchorless PrP constructs has been reported by others and is likely due to ER-associated degradation of the proteins after their synthesis (Yoo *et al*., FASEB J. 39:e71016, 2025; Hizume *et al*., BBRC 391:1681–1686, 2010). Our own experiments confirm this conclusion, as described below. We have also noted this limitation in the Discussion section (lines 268-273).

To express anchorless PrP we utilized constructs in which a stop codon was introduced at serine 230 (the GPI attachment site) of WT or G126V PrP to produce constructs designated WT.S230X and G126V.S230X. We created stably transfected populations of C1.KO N2a cells expressing these constructs. Confluent cultures were grown in reduced serum (2% FBS) for 3 days. The medium was then collected, and the methanol precipitated proteins were analyzed by immunoblotting for PrP. In parallel, the cells were lysed and subjected to immunoblotting. We found that anchorless PrP was barely detectable in cell lysates, and was undetectable in the medium, even when analyzing 20 or 90 µg of protein **(see Reviewer Figure)**. This limitation on expression of anchorless forms of PrP would be exacerbated by the low-density culturing conditions we use for plating hippocampal neurons.

Reviewer #3:

"However, the membrane-anchoring effects are inferred only based on microscopical images, which are suggestive but not definitive. Controls expressing anchorless variants of wild-type PrP and its known protective polymorphisms would be needed to make this study truly convincing. While it may be possible to predict the outcome of such experiments, it would still be worthwhile to include such controls."

Response:

See response above to reviewer #2.

PART III – MINOR ISSUES

Reviewer #1:

"While the background and the aim of the study is well described in the introduction, the abstract requires revision. In line 17-20, page 2, the authors summarize the background of the study: "However, these studies did not distinguish the source of the PrP^{Sc} that activates the signaling pathway: extracellular PrP^{Sc} bound to PrP^{c} on the neuronal surface, or membrane-anchored PrP^{Sc} generated by the PrP^{c}-PrP^{Sc} conversion process." The phrase lacks clarity in articulating the primary aim of the study and the term "source of the PrP^{Sc}" is confusing. The primary aim of the study, as I understand it, is to determine whether synaptotoxicity is directly associated with PrP conversion or occurs

independently of it. On page 5, line 87, the authors phrase the objective of the study in a clearer way: "To determine whether conversion of cell-surface PrP^{c} to PrP^{Sc} is essential for prion neurotoxicity, we have employed two different strategies." (Minor)

Response:

To improve the clarity of the abstract, we have changed the relevant sentence to read: "However, these studies did not determine whether synaptotoxic signaling is directly linked to conversion of cell-surface PrP^{C} to PrP^{Sc}, or whether it can be initiated by extracellular PrP^{Sc} independently of PrP conversion." (Lines 18-20)

Reviewer #2:

"While N2a-KO and CAD5-KO cells demonstrate that the G126V and V208M mutations do not support prion replication, it remains unclear whether cultured hippocampal neurons share similar responses to these strains. To reinforce this point, the authors should provide evidence that these prion protein variants do not support prion replication in hippocampal neurons as well. If western blotting is limited by sample availability, immunohistochemistry (IHC) or other sensitive detection methods (RT-QuIC or PMCA) could be used to confirm the accumulation of PrP^{Sc}."

Response:

Fig. 3 demonstrates, using immunofluorescence staining of membrane-associated PrP^{Sc} puncta, that rapid conversion of cell surface PrP^{C} to PrP^{Sc} is required for prion synaptotoxicity. However, we agree with the reviewer that a biochemical experiment demonstrating lack of sustained prion propagation in G126V PrP-expressing neurons would strengthen the argument. We have therefore performed the additional experiment shown in new Fig. S7, which demonstrates that G127V PrP is unable to sustain chronic prion propagation in cortical-hippocampal neuronal cultures based on analysis of PrP^{Sc} by western blotting. The combination of the immunofluorescence experiment in Fig. 3 and the biochemical experiment in Fig. S7 confirms that the G126V PrP mutant is unable to mediate synaptotoxic signaling (Fig. 2) because it is conversion-incompetent under both acute and chronic infection paradigms. We have added a new paragraph to the Results section to describe the experiment in Fig. S7 (lines 178-187).

"2. Regarding the second experimental strategy, the use of a cross-species (mouse-hamster) models with a strong species barrier, likely preventing prion replication due to incompatibility between the incoming prion seeds and the recipient PrP. Therefore, the absence of newly synthesized PrP^{Sc} under these conditions cannot be taken as evidence that synaptotoxicity requires membrane-bound prions".

Response:

The reviewer is correct that the 11 mismatches in the amino acid sequences of mouse and hamster PrP will impede cross-conversion. However, as we state in the Discussion, there is evidence that "mouse PrP^{Sc} will bind to hamster PrP^{C} and vice versa, even though

conversion is inhibited by the mismatch in PrP amino acid sequence between the two species [39, 41]". Thus, the experiment shows that simply capturing exogenous PrP^{Sc} on the cell surface via binding to PrP^{C} is insufficient to generate a synaptotoxic signal, consistent with the conclusion that endogenous, membrane-anchored PrP^{C} must be converted to PrP^{Sc}, which is the entity that generates the signal. As stated in the same paragraph of the Discussion, the same argument is likely to apply to the conversion-resistant mutants (G126V and V208M), since these amino acid substitutions lie outside of the known PrP^{C}-PrP^{Sc} binding sites. Thus, the two strategies we have adopted in our study are complementary: In one, we introduce a mutation in the PrP^{C} substrate that renders it conversion-resistant, and in the other we use a PrP^{Sc} seed from a species that cannot catalyze conversion of a non-cognate PrP^{C} substrate.

<u>Reviewer #3</u>:

"In the introduction the authors write "… the removal of the GPI anchor of PrP^{C} fundamentally alters prion pathogenesis, even though the unanchored PrP^{C} can still function as a substrate to bind and propagate PrP^{Sc}. Taken together, these previous studies suggest that PrP^{C} on the neuronal cell surface is essential for generating a neurotoxic signal in response to exogenous PrP^{Sc}." However, a study by Stöhr et al. (2011) demonstrated the spontaneous emergence of PrP^{Sc} in mice overexpressing anchorless PrP, including neurotoxic effects. Therefore, these statements would need to be moderated."

Response:

While it is true that the study by Stöhr et al., PNAS (2011) demonstrated neurotoxic effects of short protease-resistant PrP fragments in mice overexpressing anchorless PrP, this study showed that these mice developed a late-onset and spontaneous neurological disease only after ~500-600d of age. This is well above the typical incubation period of C57BL6 mice that express the anchored version of PrP and inoculated with PrP^{Sc} which is around ~120-150 days post inoculation.

We believe that the toxic effects demonstrated by these transgenic mice are due to the **chronic effects** of spontaneously generated prions in these mice. Our paper highlights a separate phenomenon by focusing on the **acute or initial effects** of prions in a cell culture system by focusing on prion conversion, the initial event of prion pathogenesis.

"On line 179 the jargon term "primary sequence" is used. It should be replaced by the proper term "primary structure".

Response:

The term "sequence" has been replaced by "structures" **(Line 191)**.

"On lines 181-182 the species barrier between mouse and hamster PrP is attributed to the 12 residues difference in the primary structure. However, in the very next paragraph it is stated that murine N2a cells transfected with hamster PrP still cannot propagate

hamster prions (263K) and that other factors seem to be at play. This apparent mismatch in statements needs to be straightened out.”

Response:

We recognize that this might be confusing.

Indeed, it is true that the species barrier phenomena arises because of the amino acid mismatches between mouse and hamster PrP sequences. This has been proven with transgenic mice that express hamster PrP are highly susceptible to hamster prions whereas WT mice are resistant (Scott et. al, Cell (1989)). However, previous studies (Bourkas et. al, JBC (2019)) have demonstrated that hamster prion strains require other cellular factors to propagate in cultured cell lines.

Our experiments further validate this finding as hamster prions seem to require both: (1) sequence homology, and the (2) appropriate cellular factors that allow them to faithfully propagate in cultured cell lines.

“The actin loading control in Figure 1D looks a bit uneven / not convincing.”

Response:

The experiment in Fig. 1D has been repeated and replaced with a new panel that shows more consistent actin staining **(Figure 1D).**

“In Figure 4A it would make it easier to understand the figure if the vertical labels “D18 (PrP)” and “3F4 (PrP)” would be swapped.”

Response:

The ordering of the vertical labels “D18 (PrP)” and “3F4 (PrP)” have been swapped **(Figure 4A)**

“In Figure S2 the labels “N2Ap” (panel B) and “N2ap” (panel E) are not defined. Presumably they refer to N2A parent (mentioned in panel D).”

Response:

We have relabeled all the N2ap lanes as N2a parent, which is now defined in the legend (**Lines 596 to 598)**

“Figure S5A shows glia cells which are not mentioned in the figure legend.”

Response:

We have added the following sentence to the legend clarifying the use of glial cells: The glial cells in the feeder layer (consisting of astrocytes) were plated, transduced, and imaged as a negative control to check for neuron-specific transduction of eGFP.

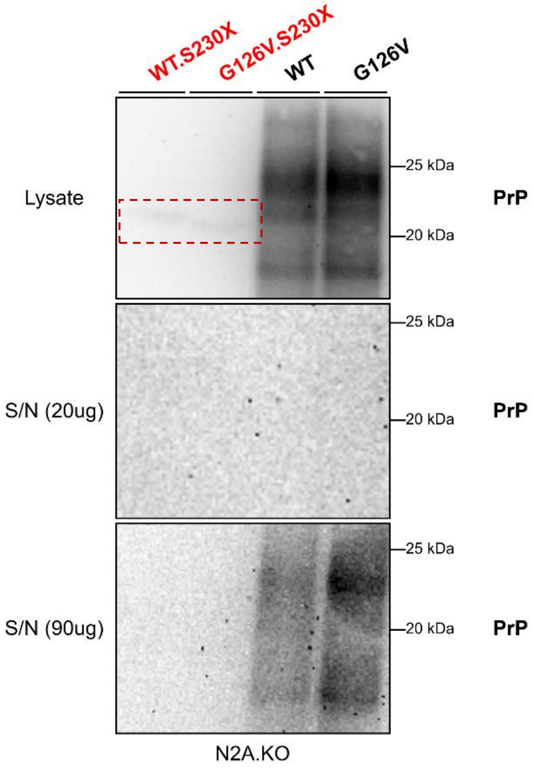

Reviewer Figure: Low/Undetectable levels of anchorless PrP in lysates and media of cultured cell lines

Prnp$^{-/-}$ N2a cells (C1.KO) expressing GPI-anchored PrP (WT or G126V) or anchorless PrP (WT.S230X or G126V.S230X) were grown to confluence, and then cell lysates and media (20 or 90 µg of protein) were analyzed by western blotting for PrP. GPI-anchored PrP is easily detectable in cell lysates and in media (as a result of α-cleavage by ADAM10 metalloprotease). In contrast, anchorless PrP is barely detectable in cell lysates (dashed red box), and is undetectable in media. The anchorless PrP in cell lysates is incompletely glycosylated, reflecting its processing via the ER-associated degradation pathway.
